# Screening preschool children with toothache: validation of the Brazilian version of the Dental Discomfort Questionnaire

**DOI:** 10.1186/1477-7525-12-30

**Published:** 2014-03-04

**Authors:** Anelise Daher, Judith Versloot, Cláudio Rodrigues Leles, Luciane Rezende Costa

**Affiliations:** 1 Health Sciences Graduate Program, Federal University of Goias, Goiania, GO, Brazil; 2 Department of Adolescent Medicine, Hospital for Sick Children, University of Toronto, Toronto, ON, Canada; 3 Division of Prosthetic Dentistry, Faculty of Dentistry, Federal University of Goias, Goiania, GO, Brazil; 4 Division of Pediatric Dentistry, Faculty of Dentistry, Federal University of Goias, Goiania, GO, Brazil; 5 Faculdade de Odontologia, Primeira Avenida, Setor Universitario, Goiania, GO, Brazil

**Keywords:** Toothache, Child, Preschool, Pain measurement, Validation studies

## Abstract

**Background:**

The Dental Discomfort Questionnaire (DDQ) is an observational instrument intended to measure dental discomfort and/or pain in children under 5 years of age. This study aimed to validate a previously cross-culturally adapted version of DDQ in a Brazilian children sample.

**Methods:**

Participants included 263 children (58.6% boys, mean age 43.5 months) that underwent a dental examination to assess dental caries, and their parent that filled out the cross-culturally adapted DDQ on their behalf. Exploratory factor analysis (principal component analysis form) and psychometric tests were done to assess instrument’s dimensionality and reliability.

**Results:**

Exploratory factor analysis revealed a multidimensional instrument with 3 domains: ‘eating and sleeping problems’ (Cronbach’s alpha 0.81), ‘earache problems’ (alpha 0.75), and ‘problems with brushing teeth’ (alpha 0.78). The assessment had excellent stability (weighted-kappa varying from 0.68 to 0.97). Based on the factor analysis, the model with all 7 items included only in the first domain (named DDQ-B) was further explored. The items and total median score of the DDQ-B were related to parent-reported toothache and the number of decayed teeth, demonstrating good construct and discriminant validities.

**Conclusions:**

DDQ-B was proven a reliable pain assessment tool to screen this group of Brazilian children for caries-related toothache, with good psychometric properties.

## Abstract (pt)

**Portuguese abstract:**

**Proposição**

O *Dental Discomfort Questionnaire* (DDQ) é um instrumento observacional usado para avaliar dor de dente/desconforto em crianças menores de 5 anos de idade. Este estudo objetivou validar uma versão brasileira do DDQ, previamente adaptada transculturalmente.

**Métodos**

263 crianças participaram do estudo (58.6% meninos, com idade média de 43,5 meses), as quais foram examinadas clinicamente para avaliar a ocorrência de cárie, e seus pais preencheram individualmente a versão brasileira do DDQ. Para avaliar a dimensionalidade e confiabilidade do instrumento, foram realizados análise fatorial exploratória (tipo: análise de componentes principais) e testes psicométricos.

**Resultados**

A análise exploratória fatorial revelou um instrumento multidimensional com 3 domínios: ‘problemas durante a mastigação e sono’ (alfa de *Cronbach* 0,81), ‘problemas relacionados à dor de ouvido’ (alfa 0,75), e ‘problemas durante a escovação’ (alfa 0,78). O instrumento mostrou excelente estabilidade (kappa ponderado variando de 0,68 a 0,97). Baseado nos resultados da análise fatorial exploratória, o modelo com os 7 itens incluídos no primeiro domínio, denominado DDQ-B, foi adicionalmente explorado. A frequência dos itens e o escore total do DDQ-B associaram-se a dor de dente relatada pelos pais das crianças e ao número de dentes cariados, confirmando as validades de construto e discriminante.

**Conclusão**

O DDQ-B mostrou-se confiável e com boas propriedades psicométricas para avaliar este grupo crianças brasileiras apresentando dor de dente por cárie.

## Background

Pain, in general, is most reliably measured using self-report, when available, given that pain is a subjective experience [1]. Assessing pain in preschoolers and early-verbal children, however, presents special challenges, as their cognitive capacities are still under-developed. As a result young children would describe pain in global and emotional terms and would have difficulties in perceiving, understanding, remembering and reporting pain [2]. In addition, this cognitive immaturity often makes it difficult for them to communicate verbally and, consequently, to reliably self-report their pain [3,4].

To avoid the inaccurate assessment of pain in very young children, it is recommended to use a validated observation tool that assesses pain based on the observation of pain-related behaviors [5]. Alternatively, parents can give a proxy report on children’s pain, as it has been demonstrated that children’s pain as perceived by their parents is correlated with their self-report of pain [6,7]. Unfortunately, proxy reports of a child’s pain by their parents or healthcare provider is often not exact. Both over and underestimations of proxy reported pain of children are reported in the literature resulting in suboptimal care [8,9].

Recognizing toothache in preschool children is similarly inherently difficult. The tissue damage related to dental caries, which often causes toothache, is not obvious to parents. Consequently, parents regularly do not realize that their child has a toothache. Furthermore, the behavioral expression of children as a result of toothache is often thought by parents to be related to earache, a type of pain that is more familiar to them. Dental caries, a disease that can result in toothache, however, is one of the most prevalent infectious diseases among preschool children worldwide. For example: a recent study among 4-5-year-old Chinese children showed a prevalence of 72% of caries in primary teeth [10]; among 2-5-year-old American children an increase of caries prevalence was found from 23% during the period 1998-1994 to 28% during the period 1999-2004 [11]; and the last national survey of 5-year-old Brazilian children revealed a prevalence of dental caries of 53.4% [12]. The occurrence of caries in children is considered to be an important predictor of the onset of pain. One in five children with decayed teeth (teeth with cavity due to caries) present with toothache [13]. Furthermore, caries in preschoolers is associated with lower quality of life due to the effects of pain [14]. For this reason it is of great importance that toothache is recognized in young children and that appropriate treatment is sought to eliminate the caries and the associated toothache. Besides using proxy reports to assess toothache in this age group a general pain assessment tools could be used [12,13,15,16]. These general tools, however, are not focused on specific behaviors that children can present as a result of having toothache and consequently toothache might stay unrecognized. Therefore, it is better, when expecting toothache, to use a specific observational instrument that focusses on toothache related behaviors such as the Dental Discomfort Questionnaire.

The Dental Discomfort Questionnaire (DDQ) is a behavioral observation tool developed to recognize toothache in children aged 5 years or younger, which focuses on toothache-related pain behaviors [17]. The DDQ was developed in The Netherlands based on two concepts: 1) caries and toothache in young children often results in e.g., problems with eating, sleeping and brushing teeth [15,16] and 2) children’s abilities to verbalize pain depends on their developmental cognitive stage [4]. To get an insight into which behaviors children with toothache often display and to see if the presence of these particular behaviors could be used to identify toothache, experienced dentists interviewed parents of children with toothache to ask them about the behaviors of their children. These interviews resulted in a list of 12 behavior items for which the prevalence was tested in a group of young children [17]. Eight out of the 12 behaviors in the list were found to be more often present in children with caries and toothache than in children without caries and toothache. Three items concerning earache and one item concerning sleeping problems occurred frequently in both groups of children and then were removed [17]. Those 8 items formed the DDQ. Psychometric properties of the 8-item DDQ tested on preschoolers showed satisfactory internal consistency (alpha 0.74) [17], as well as toothache prediction ability (ROC area 0.88, CI 0.81-0.94). A score of 3 or higher identified 78.0% of children with toothache in that study [18]. After children undergone dental treatment the total score of the DDQ significantly reduced in comparison with both before and immediately after treatment [19] and after an 8 week follow-up period [20], further supporting the validity of the DDQ. Finally, the developers of the DDQ also developed a version for children with learning disabilities [21]. Overall, the DDQ can be categorized as an ‘approaching well-establishment’ pain tool [1,3], but its dimensionality has never been tested through factor analysis.

To make the DDQ a ‘well-established’ pain tool it is essential to do additional studies. Exploring the DDQ’s dimensionality by means of factor analysis and making the DDQ adaptable to different cultures is of particular importance in the evidence-based process for improving the instrument establishment [1,3]. Besides, toothache assessment studies are relevant because of the known impact toothache has on preschoolers’ quality of life and the possible inherent inability of a child in this age group to express it.

The aim of this study was to validate a previously cross-culturally adapted Brazilian version of the DDQ for caries-related toothache assessment in Brazilian preschoolers.

## Materials and methods

**Figure 1:**
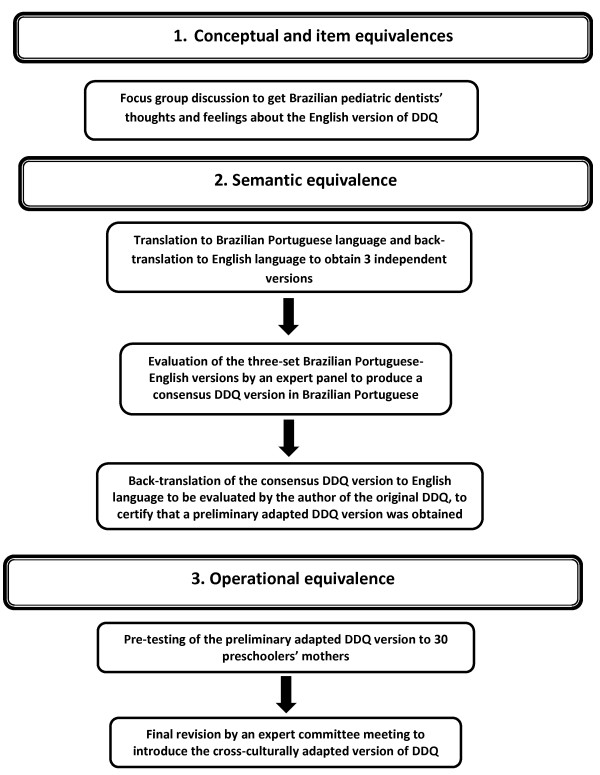
**Flowchart of cross-cultural adaptation.** Flowchart of the universalistic approach method used to cross-culturally adapt the Dental Discomfort Questionnaire (DDQ), Brazilian-Portuguese version.

The study described in this paper is the second phase of a larger study. In the first phase described in detail elsewhere (manuscript submitted to publication), the DDQ with 12-items was cross-culturally translated and adapted to be used in Portuguese-Brazil-speaking children’s caregivers, according to a universalistic approach method [22]. Figure 1 depicts this cross-cultural adaptation phase, including conceptual and item equivalences, semantic equivalence and operational equivalence. Phase two is related to the measurement and functional equivalences that are addressed in the current study (validation process). The second phase did not involve participants included in the first phase of this study.

This study was independently reviewed and approved by the Institutional Research Board of the Federal University of Goias, city of Goiania, State of Goias, Brazil (protocol #127/09). Consent was sought from all participants (parents on behalf of their children): after they understood the aims, risks, benefits and other characteristics of this investigation, they signed a written consent form to participate in this study. All phases of this study were done in full accordance with ethical principles, including the World Medical Association Declaration of Helsinki [23].

### Participants

Participants in this study included 263 children between the ages of 25 and 60 months. Participants were approached during public health services for children, including hospital outpatient clinics, dental clinics and day care centers. Children did not have any mental or physical disabilities and cooperated with the dental examination.

### The instrument: Dental Discomfort Questionnaire - DDQ

The DDQ is an observational instrument for assessing dental discomfort and/or pain in very young children [17]. The DDQ contains questions to be completed by a child’s parent or caregiver. The respondents were first asked how often their child had a toothache. Potential responses included ‘never’, ‘sometimes’, ‘often’ and ‘I do not know’. If s/he noticed that the child had a toothache, the respondent reported when this occurred, i.e., ‘during meals’, ‘during the day’ or ‘during the night’. The second part of the complete version of the original DDQ includes 12 items about different child behaviors that could be associated with toothache or dental discomfort, which are answered on a 3-point scale, as follows: 0 ‘never’, 1 ‘sometimes’, and 2 ‘often’. The twelve items included in the complete version of the DDQ are as follows: 1. ‘Bites with molars instead of front teeth’; 2. ‘Puts away something nice to eat’; 3. ‘Cries during meals’; 4. ‘Has problems with brushing lower teeth’; 5. ‘Has problems with brushing upper teeth’; 6. ‘Has earache during the day’; 7. ‘Has earache at night’; 8. ‘Has earache during eating’; 9. ‘Has problems chewing’; 10. ’Chews on one side’; 11. ‘Reaches for the cheek while eating’; and 12. ‘Suddenly cries at night’.

### Procedures

The cross-culturally adapted version, which included 12 items, was completed by parents or guardians without interference from the interviewer while their children had a dental exam. One of three pediatric dentists examined children’s dentition status following the World Health Organization (WHO) recommendations [24], after training and calibration. A convenience random sample of 14 children was dentally reexamined in a one-week interval by the three pediatric dentists to determine the intra-examiner agreement. Inter-examiner agreement was measured through pictures: The three pediatric dentists examined 12 photographs showing teeth of children under 5-years old, for 1 minute and registered the affected teeth according to the WHO decayed, missing, filled tooth index (dmft) (*in lux* calibration). Intra and inter-examiner agreement regarding dmft index were tested with kappa. The intra-examiner kappa varied from 0.82 to 0.98 for the three pediatric dentists. Taken the dentists’ results in pairs, inter-examiner reliability varied from 0.76 to 0.90.

The examiner used a mouth mirror, a WHO periodontal probe, an artificial LED head lamp (Microdont Star Light KD 200, Sao Paulo, Brazil) and personal protective equipment. Children were examined while seated in a chair or in their parents’ laps (small children), with the examiner seated in front of the chair. Dentition status was determined using the WHO caries diagnostic criteria (dmft index) [24]. Dental codes were written down on a spreadsheet by a recording clerk. Children with one or more teeth scored as ‘decayed’ or ‘filled with additional decay’ were considered to have decayed teeth.

### Statistical analysis

All statistical analyses were carried out using the IBM Statistical Package for Social Science 19.0 (SPSS Inc., Chicago, IL, USA). Statistical significance was set at *P* < 0.05.

#### ***Test-retest reliability***

For the test-retest reliability assessment, we calculated the sample size using the *2 times k*^2^ formula, where *k* is the number of points on the scale [25]. As the DDQ is a three-point scale, k = 3. According to the formula (2 times “3 squared”), a sample of 18 individuals would be sufficient for this specific test. We included a convenience sample of 38 parents to complete again the adapted version of the DDQ (with 12-items) one week after the first questionnaire was administered. The test-retest reliability coefficients of each DDQ item were calculated using the weighted-kappa [26].

#### ***Exploratory factor analysis and internal consistency***

The dimensionality of the adapted 12-item version was assessed through exploratory factor analysis: principal component analysis (PCA) form with orthogonal varimax rotation. Bartlett’s Test was used to assess sphericity and the Kaiser-Meyer-Olkin was used to measure of Sampling Adequacy (MSA) [27]. The number of factors in the instrument was reduced by using the Kaiser criterion (Eigenvalues over 1.0) [27]. The varimax rotation method was chosen to minimize the likelihood of two or more significant factors loading for each item by maximizing the extent to which factors were independent of each other [28]. Item values were retained if they had a primary factor loading of >0.40 and a secondary factor loading of <0.30 [29]. After the PCA, the internal consistency of the different factors was assessed using Cronbach’s alpha [30]. We expected to find two factors: one related to earache problems (3 items) and other comprising the other 9 items.

#### ***Construct and discriminant validity***

Construct validity was tested by associating the median total score of children with and without parent-reported toothache (dichotomized as yes = ‘sometimes’ and ‘often’, and no = ‘never’) (Mann-Whitney U Test) and correlating it with the number of decayed teeth (Spearman correlation). For discriminant validity, frequencies of the pain-related behaviors (DDQ items) and the median of DDQ total score were compared between four clinical groups of children, divided accordingly to the occurrence of caries and toothache reported by parents [31].

## Results

### Participants

Participants in this study included 263 children, 109 girls and 154 boys, between the ages of 25 and 60 months (mean 43.5, SD 9.8), and their parents who completed the questionnaires. Most questionnaires were filled out by mothers (84.8%), followed by fathers (9.5%) and grandmothers (5.7%).

### Test-retest reliability

The test-retest reliability of the 12-item adapted DDQ using a weighted-kappa for all items showed an excellent stability for most items of the instrument (Table 1).

**Table 1:** Factor loadings after varimax rotation of the three components extracted and results of test-retest reliability for each DDQ item

**DDQ items**^**(a)**^	**Factor 1–‘Eating and sleeping problems’**	**Factor 2–‘Earache problems’**	**Factor 3–‘Problems with brushing teeth’**	**Weighted-kappa coefficient (95% Confidence Interval)**^**(b)**^
Cries during meals	**0.79**	-0.26	-0.09	0.88 (0.74-1.00)
Reaches for the cheek while eating	**0.77**	0.24	0.06	0.75 (0.55-0.96)
Puts away something nice to eat	**0.70**	0.01	0.16	0.74 (0.52-0.96)
Suddenly cries at night	**0.60**	0.24	0.27	0.84 (0.69-0.98)
Chews on one side	**0.59**	0.28	0.17	0.97 (0.92-1.00)
Has problems chewing	**0.53**	0.20	0.36	0.77 (0.61-0.94)
Bites with molars instead of front teeth	**0.47**	-0.08	0.30	0.68 (0.49-0.88)
Has earache during the day	0.04	**0.86**	0.07	0.80 (0.59-1.00)
Has earache at night	0.05	**0.75**	0.07	0.94 (0.84-1.00)
Has earache during eating	0.22	**0.72**	0.07	0.94 (0.83-1.00)
Has problems with brushing lower teeth	0.07	0.09	**0.87**	0.86 (0.71-1.00)
Has problems with brushing upper teeth	0.26	0.11	**0.84**	0.75 (0.57-0.93)
Eigenvalues	4.08	1.62	1.28	
% of variance	34.04	13.52	10.67	
Cronbach’s alpha coefficient	0.81	0.75	0.78	

^(a)^Items ordered by factor analysis. ^(b)^Test-retest reliability.

The numbers in bold refer to the highest factor loading for each item.

### Exploratory factor analysis and internal consistency

A total of 211 questionnaires were included for exploratory factor analysis, principal component analysis (PCA) form. In 52 cases, parents did not complete the 12 items of the survey; therefore, these questionnaires were excluded from further analysis. The remaining questionnaires showed good sampling adequacy and sphericity for correlation assessment among the items to proceed with the PCA. Indeed, Bartlett’s Test of Sphericity showed a significant correlation among items (*P* < 0.001), and the Kaiser-Meyer-Olkin Measure of Sampling Adequacy (MSA) reached a value of 0.77.

Next, the factors using PCA with orthogonal varimax rotation were extracted. Of the 12 possible factors extracted, three were considered relevant (Eigenvalues > 1). This model explained 58.2% of the total variance. Table 1 presents the factor loadings for each item after varimax rotation. The first factor aggregated items about functions related to mastication and possible discomfort during eating or sleeping and was called the ‘eating and sleeping problems’ domain. The second factor addressed earache, which was reported in different periods; this was called the ‘earache problems’ domain. Finally, the third factor focused on problems brushing one’s upper or lower teeth and was called the ‘problems with brushing teeth’ domain.

The reliability analysis for each extracted factor showed that the questionnaire with items from the first domain (‘eating and sleeping problems’) had a good internal consistency (Cronbach’s alpha coefficient 0.81), while the other two domains had acceptable Cronbach’s alpha, 0.75 and 0.78 respectively (Table 1) [29]. Alpha coefficients could not be improved by the exclusion of any item, and all 12 items had good convergent validity (item scale correlation ≥ .40).

The first factor of DDQ was named DDQ-Brazil (DDQ-B) and was further explored. The other two factors were not considered for the additional analyses.

### Construct and discriminant validity analysis of DDQ-B

In response to the toothache question, parents reported that 46.4% (n = 122) of their children ‘never’ had toothache, 38.4% (n = 101) had such pain ‘sometimes’, 9.9% (n = 26) had such pain ‘often’, and the parents of 14 (5.3%) did not know. Parents of participating children reported that 42.3% (n = 111) had toothache during eating, 40% (n = 105) had it during the day, and 20% (n = 52) had toothache at night. Sixty percent of children had decayed teeth (n = 158). Regarding children with reported toothache, parents reported more toothache (‘sometimes’ and ‘often’) for children with decayed teeth (n = 112, 88.2%) than for children without decayed teeth (n = 15, 11.8%) (*P* < 0.001, Chi-square test). Four clinical groups based on the combination of decayed teeth and toothache reported by parents (excluding the questionnaires with the ‘did not know’ marked option for toothache question, n = 14) were identified: children with decayed teeth and toothache (children with caries-related toothache) (group 1, n = 112); children with decayed teeth but no toothache (group 2, n = 46); children without decayed teeth but with toothache (group 3, n = 15); and children without decayed teeth or toothache (children without caries-related toothache) (group 4, n = 76).

The median total score on the DDQ-B was 2.0 (first-third quartile 1.0-5.0), and scores ranged from 0 to 13. The children’s ages did not correlate with the total score (Spearman’s *rho* = 0.08, *P* = 0.17). Boys had higher median total scores (3.0, first-third quartile 1.0-6.0) than girls (2.0, first-third quartile 0.5-4.0) (*P* = 0.01, Mann-Whitney test); there were no differences between boys and girls in toothache reported by parents (*P* = 0.71, Chi-Square test).

Children whose parents reported toothache presented with higher total DDQ-B scores (median 4.0, first-third quartile 2.0-7.0) than those with no toothache (median 1.0, first-third quartile 0.0-3.0) (*P* < 0.001, Mann-Whitney test). Moreover, children with higher DDQ-B scores had a higher number of decayed teeth (Spearman’s rho = 0.42, P < 0.001).

The clinical groups (1, 2, 3 and 4) were characterized by different frequencies of pain-related behaviors (*P* < 0.05, Kruskal-Wallis test) and different median total DDQ-B scores (*P* < 0.001, Kruskal-Wallis test). The seven pain-related behaviors and the median total DDQ-B scores are displayed in Table 2 and show the discriminant validity of DDQ-B in identifying children with decayed teeth and toothache. The median total DDQ-B score of children with decayed teeth and toothache was higher than the median total scores of children included in other groups (*P* < 0.001, Mann-Whitney test). Moreover, children with decayed teeth and toothache (group 1) exhibited all of the pain-related behaviors on DDQ-B more often than caries-free children and those without parent-reported toothache (group 4) (*P* < 0.01, Mann-Whitney test). There was no difference between children with decayed teeth without toothache and children without decayed teeth with toothache (groups 2 and 3) in any of the individual items or median total scores.

**Table 2:** Frequency of ‘sometimes’ and ‘often’ for each Dental Discomfort Questionnaire–Brazilian version (DDQ-B) item and DDQ-B total scores for different clinical groups

**Pain-related behaviors from the DDQ-B (‘sometimes’ and ‘often’)**	**n (%)**			
	**Children with decayed teeth and a toothache**	**Children with decayed teeth without a toothache**	**Children without decayed teeth with a toothache**	**Children without decayed teeth or a toothache**
Bites with molar instead of front teeth	70 (62.5)^a^	27 (60.0)^a^	8 (53.3)^a,b^	33 (43.4)^b^
Puts away something nice to eat	58 (51.8)^a^	12 (26.1)^b^	1 (6.7)^b^	12 (15.8)^b^
Crying during meals	68 (60.7)^a^	8 (17.4)^b^	3 (20.0)^b^	10 (13.2)^b^
Problems chewing	61 (54.5)^a^	12 (27.3)^b^	2 (13.3)^b^	11 (14.5)^b^
Chewing on one side	54 (61.4)^a^	12 (34.3)^b^	3 (23.1)^b,c^	10 (15.9)^c^
Reaching for the cheek while eating	68 (60.7)^a^	9 (20.4)^b^	4 (26.7)^b^	8 (10.5)^b^
Suddenly cries at night	64 (57.1)^a^	16 (36.4)^b^	7 (46.7)^b^	21 (27.6)^b^
Median total DDQ-B score (*first-third quartile*)	5.0 (2.5-7.0)^a^	2.0 (1.0-4.0)^b^	2.0 (1.0-3.5)^b,c^	1.0 (0-2.0)^c^

The same letter indicates an insignificant difference, whereas different letters indicate significant differences (P < 0.05) among groups.

## Discussion

The psychometric characteristics of a cross-culturally adapted version of the Dental Discomfort Questionnaire (DDQ) to the Brazilian culture were satisfactory. A shorter model of DDQ with 7 items, named DDQ-B, was further explored and validated as a preschooler toothache assessment tool, presenting adequate psychometric and discriminant properties. The ability to discriminate between the presence and absence of pain is the most important quality of any pain assessment measure [32].

The initial 12-item Brazilian Portuguese adaptation of the DDQ was found to be a multidimensional instrument with three clearly distinct domains: ‘eating and sleeping problems’, ‘earache problems’ and ‘problems with brushing teeth’. Previous study [17] affirmed that the DDQ could be seen as a one-dimensional scale, although that statement was based on its satisfactory internal consistency only and not on factor analysis. Our aprioristic expectation of two factors was partially confirmed; however, a third “unexpected” factor included the two items related to “tooth brushing problems”, and we understand that they might be observed in stubborn young children that might have not been in pain. Exploring each factor individually, the ‘eating and sleeping problems’ domain, which included 7 items, showed the highest internal consistency. Moreover, this domain on its own demonstrated a high test-retest reliability, which supports excellent stability. Together, these findings suggest that the 7-item DDQ-B has good psychometric properties. The repeated psychometric analyses described in this study strengthen the properties of the DDQ. Analyses performed in different situations/research groups are criteria for a “well-established” pain assessment tool for use with children in clinical settings [1,3].

Pain-related behaviors are important indicators for the assessment of pain in preverbal children [6]. As stated in our results, pain-related eating and sleeping behaviors were found to be more frequently displayed by children with decayed teeth and toothache than in children who were caries-free and without toothache. In another study, it was also found that problems eating certain foods, as reported by parents, were more prevalent in young children with decayed teeth than without [15].

Interestingly, we found that boys had higher DDQ-B scores than girls, but there were no significant sex differences found in the incidence of toothache as reported by parents. This result might be due to the interplay between pain expression, socialization and gender [33]. The presented study focused on preschoolers. In this age range, boys may still be more emotionally expressive than girls; however, at around six years old, boys become less likely than girls to express hurt or distress, as they are made to feel ashamed of their feelings of weakness [34].

One limitation of this study is that the gold standard measure of toothache was parental report. This could potentially result in an imperfect reference standard [35]. To minimize this bias, the occurrence of caries was also assessed and was found to be associated with the presence of toothache. Parents reported more toothache when children had decayed teeth (caries) than when they did not. To further eliminate bias for parts of our analysis we chose to exclude the children who had caries but for whom the parents indicated they did not have toothache (possible false negatives) and the children who did not have caries but for whom the parents indicated they did have toothache (possible false positives). A previous cohort study similarly showed that toothache is rare in caries-free children but common in children who had caries [13]. The use of different methods in a gradual process of validation with confirmation of the meaning of the analyzed data is recommended [35]. This approach was done for construct and discriminant tests in an attempt to improve parent-reported toothache as the gold standard as for very young children, self-reporting could be misinterpreted [5].

One way to address the lack of a true gold standard was to conceptualize pain as a latent variable (one that cannot be observed) and use the results of the present exploratory factor analysis to generate hypotheses for confirmatory factor analysis [27,31]. Therefore, further studies with larger samples should be conducted in the future to estimate the measurement error when analyzing the relationships between DDQ items.

Furthermore, the item ‘bites with molars instead of front teeth’ should be viewed with caution, as it does not discriminate between children with toothache. It was kept in the DDQ-B because its removal would not improve the Cronbach’s alpha. It may be that parents have difficulties observing whether a child is biting with the front or back teeth.

The DDQ-B offers unique benefits in the assessment of toothache and the subsequent prioritization of dental treatment among Brazilian children. For the assessment of toothache, there is only one validated Brazilian instrument (the Child Dental Pain Questionnaire, child-DPQ) with good psychometric properties (ICC = 0.99 and Cronbach’s alpha 0.81), but it is used only with children between the ages of 8 and 10 years [36]. Moreover, relevant oral health epidemiological data shows children’s need for a validated instrument: more than half of Brazilian children who are 5 years of age and under have caries in primary dentition according to a recent national government study [12]. Similar high caries prevalence are found in this age group in Southern (40% of children 0 to 5 years old) [37] and Northern Brazil (62% of children 2 to 4 years old) [15]. Moreover, 25% of parents/caregivers of preschoolers in Brazil indicated their child had a history of some type of toothache in their lifetime [16].

As our sample was not representative of the Brazilian population, caution should be taken when generalizing the results. Nonetheless, the present findings adds to the literature and support an instrument that can be applied in clinical practice and in research, in private office and in public services, to help with the diagnosis of a condition that affects the quality of life of children worldwide. Yet, our results indicate that the original DDQ psychometric properties should be reassessed in English-speaking cultures.

In summary, the 7 items-model of the Brazilian version of the Dental Discomfort Questionnaire (DDQ-B) is a reliable and validated toothache assessment tool for screening caries-related toothache among 2- to 5-year-old preschool children and has good psychometric properties. Children’s eating- or sleeping-related problem behaviors would seem to be the most suitable areas of concern for toothache assessment purposes and should be systematically investigated. Finally, the presented methodology sequence could be used in future research to adapt the DDQ and other observational instruments to other cultures and languages.

## Competing interests

The authors declare that they have no competing interests.

## Authors’ contributions

AD designed and conducted the study, analyzed the data, and wrote the manuscript. JV is the DDQ developer, designed the study, analyzed the data, and wrote the manuscript. CRL contributed with the statistical knowledge, analyzed the data and wrote the final reviewed version of the manuscript. LRC was responsible for the study supervision, designed and conducted the study, analyzed the data, and wrote the manuscript. All authors reviewed and approved the final manuscript prior to its submission.
